# Occurrence and Distribution of Fasciolosis in a Cohort of Ovine Livestock Restricted to a Mountain Plateau in Central Portugal

**DOI:** 10.3390/ani11123344

**Published:** 2021-11-23

**Authors:** Catarina Coelho, Rita Cruz, Fernando Esteves, Helena Vala, Maria A. Pereira, Irina Amorim, Carmen Nóbrega, João R. Mesquita

**Affiliations:** 1 Polytechnic Institute of Viseu (ESAV), Agrarian School of Viseu, 3500-606 Viseu, Portugal; ccoelho@esav.ipv.pt (C.C.); rcruz@esav.ipv.pt (R.C.); festeves@esav.ipv.pt (F.E.); hvala@esav.ipv.pt (H.V.); mapereira@esav.ipv.pt (M.A.P.); cnobrega@esav.ipv.pt (C.N.); 2 Animal and Veterinary Research Center (CECAV), University of Trás-os-Montes e Alto Douro, 5000-801 Vila Real, Portugal; 3 Centre for Studies in Education and Health Technologies (CI&DETS), 3500-606 Viseu, Portugal; 4 Centre for the Research and Technology of Agro-Environmental and Biological Sciences (CITAB), University of Trás-os-Montes e Alto Douro, 5000-801 Vila Real, Portugal; 5 Global Health and Tropical Medicine (GHTM), Instituto de Higiene e Medicina Tropical (IHMT), 1349-008 Lisboa, Portugal; 6 ICBAS—Institute of Biomedical Sciences Abel Salazar, University of Porto, Rua Jorge de Viterbo Ferreira 228, 4050-313 Porto, Portugal; iamorim@ipatimup.pt; 7 Institute of Molecular Pathology and Immunology of the University of Porto (IPATIMUP), 4099-002 Porto, Portugal; 8 Institute for Research and Innovation in Health (i3S), University of Porto, 4099-002 Porto, Portugal; 9 Epidemiology Research Unit (EPIUnit), Instituto de Saúde Pública da Universidade do Porto, 4050-091 Porto, Portugal

**Keywords:** *Fasciola hepatica*, *Trematoda*, sheep, Portugal

## Abstract

**Simple Summary:**

*Fasciola hepatica* is a parasite that affects ruminants. This study evaluated the occurrence of fasciolosis in ovine livestock from central Portugal during a 2-year period. Positive animals were found in most locations and in both years (19.6% and 18.5% seropositive in the first year and second year, respectively). Correct anthelmintic treatment could further reduce egg elimination and pasture contamination.

**Abstract:**

*Fasciola hepatica* is a parasite that is widespread in Europe, having been reported in ruminants of several European countries and causing an important economic impact. This study ascertained the occurrence and distribution of fasciolosis in Portuguese ovine livestock by assessing *F. hepatica* IgG antibodies in a cohort of confined sheep from a high-altitude region of central Portugal in a 2-year period. Positive animals were found in most locations and in both years, with 18 of the 92 animals (19.6% [95% confidence interval CI: 12.03–19.15]) and 17 of the same 92 animals (18.5% [95% CI: 11.15–27.93]) showing to be seropositive in the first year and second year, respectively (*p* = 0.85). Pasture contamination by *F. hepatica* eggs could be reduced by thorough anthelmintic treatments.

## 1. Introduction

Zoonotic foodborne trematodiases (ZFTs) are caused by species of the genera *Clonorchis*, *Opisthorchis*, *Paragonimus* and *Fasciola*, known to cause up to 7000 deaths and 200,000 morbidity cases each year, with an estimated 2 million disability-adjusted life-years worldwide [1]. Among these ZFTs, *Fasciola* spp. parasites pose an important impact in herbivores but also occur in humans. The worldwide prevalence of *Fasciola* infection in humans is calculated to range between 2.4 and 17 million and is considered under-reported and under-diagnosed [2,3]. *Fasciola hepatica* is endemic to Asia and Europe, occurring in lower numbers in South and Central America, Middle East and Northern Africa, with sporadic cases occurring in the United States of America and the Caribbean [2]. *Fasciola gigantica* occurs in the Pacific Islands, Asia and Northern Africa [2]. Domestic ruminants are the most common definitive hosts and humans living in close proximity to cattle and sheep industries and who consume raw aquatic vegetation are particularly at risk [4], overall causing serious public health concerns and considerable economic losses [5,6]. However, studies have shown that in some regions fascioliasis in humans does not necessarily occur in areas where fascioliasis is a major veterinary problem. This typically occurs in human hyperendemic zones such as the Bolivian Altiplano, where human prevalences are sufficient and maintained over time; egg fecal shedding in humans is sufficiently high and shed eggs are proved to be viable [7,8,9].

In domestic livestock fasciolosis is an important disease [10] and both immature and mature stages of the parasite in the final host result in a 15% decrease in milk yield [11], an average reduction of 1.5 kg [12] or 0.7 kg milk/cow per day [13]. Annual losses have been estimated to be around EUR 2.5 billion to the livestock and food industry worldwide [14].

*Fasciola hepatica* is a parasite that is widespread in Europe and has been reported in ruminants of several European countries such as Belgium, Denmark, England, Germany, Ireland, Italy, Poland, Spain, Switzerland, and Wales [15].

To the best of our knowledge no study has been performed serologically ascertaining the occurrence and distribution of fasciolosis in Portuguese ovine livestock. As such we assessed *F. hepatica* IgG antibodies in a cohort of confined sheep from a high-altitude region of central Portugal in a 2-year period.

## 2. Materials and Methods

This study considered sheep from the Serra da Estrela breed, a local autochthonous breed, to best mirror the circulation of *F. hepatica*. This sheep breed is located in the Serra da Estrela mountain plateau, with vast local pastures where sheep graze. The average annual temperature is ~7 °C, with an average precipitation from 1000 mm to 2500 mm per year. This sheep breed is managed by the “National Association of Serra da Estrela Sheep breed” (ANCOSE) that assures geographical restraint to this region to maintain the breed status. As these animals are confined to this region, the assessment of their serological status can be a valuable tool to reflect the local circulation of diseases [16,17].

Sera collection was performed in a previous study [17] preserved at −80 °C. The sampling scheme consisted of sera from a sheep cohort (n = 92), initially collected in January/February 2015, and again in January/February 2016 from the same animals (in a total of 184 sera samples = 92 paired). As herd size averages 40 animals, a total of four animals (~10% of herd size) aging 6 months and older, was randomly selected from 23 herds of Serra da Estrela sheep, located on 21 parishes of 4 municipalities of the region (Carregal do Sal, Celorico da Beira, Gouveia, and Seia). All 184 sera were screened for *F. hepatica* IgG antibodies, using a commercially available enzyme-linked immunosorbent assay (IDEXX *Fasciola hepatica* antibody test kit, Hoofddorp, the Netherlands), an assay based on the coating of microwells by f2 antigen of *F. hepatica* that has been shown to present 100% sensitivity and specificity [18]. Procedures were performed according to the manufacturer’s instructions, with samples being tested in duplicates. Optical densities (OD) were measured at 450 nm. Sample to positive ratio (S/P%) were calculated and considered negative if S/P% ≤ 30% and positive if S/P% > 30%, as described by the manufacturer. To assess differences in the seropositivity obtained in each year, a Chi-square test was used (GraphPad Prism version 5.04, GraphPad Software, La Jolla California), considering *p* values < 0.05 as statistically significant.

## 3. Results

Screening for *F. hepatica* IgG antibodies revealed that positive animals were found in most locations and in both years. In particular, in the year 2015, a total of 18 of the 92 animals showed to be seropositive, while in 2016, 17 of the same 92 animals were positive. This corresponds to an occurrence of 19.6% (95% confidence interval [CI] 12.03–19.15) in 2015 and 18.5% (95% CI: 11.15–27.93) in 2016. These proportions were found to be not statistically different (*p* = 0.85), and only 1 animal seroreverted from the 1st to the 2nd year. The occurrence of anti-*F. hepatica* IgG seropositive animals in 2015 and 2016 is depicted in Table 1.

**Figure 1:**
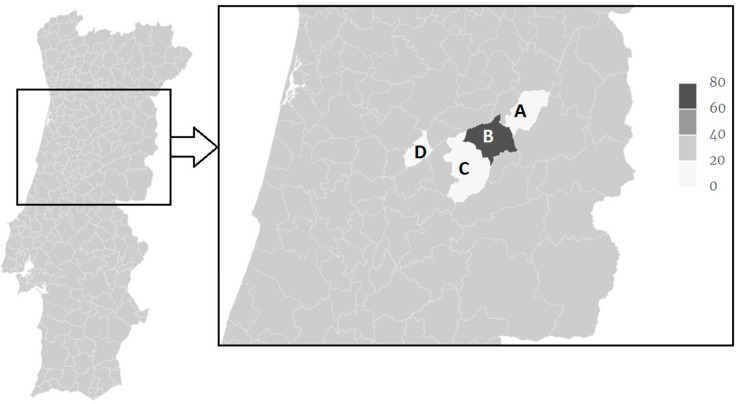
Distribution of *F. hepatica* IgG seropositivity Serra da Estrela sheep sampled in the years 2015 and 2016 in Celorico da Beira (A), Gouveia (B), Seia (C) and Carregal do Sal (D) municipalities.

A distinction between high versus low endemicity regions can also be observed (Figure 1) as the Gouveia municipality presented an occurrence of *F. hepatica* IgG seropositive animals of 62.5% in both years, while all others where below 20%. In particular, Carregal do Sal presented 0% (95% CI: 0–26.5), Celorico da Beira 3.6% (95% CI: 0.1–18.4) in both years, Seia presented 19.4% (95% CI: 6.5–32.4) and 16.7% (95% CI: 6.4–32.8) in 2015 and 2016, respectively (Table 1).

## 4. Discussion

Subacute fasciolosis in sheep is a worthy cause of poor reproductive performance and is associated with high rates of non-pregnancy, reduced twinning rates and prolonged lambing periods [19]. In cases of chronic fasciolosis, a reduction of the growth rate can be seen in young animals, while weight loss, reduction of milk production and wool quality is observed in adults [20]. Fasciolosis undermines animal health status, leading to a high morbidity and mortality [21] whereby it is necessary to prevent and to improve the diagnostic capacity.

Data on the occurrence and distribution of fasciolosis in sheep in Europe are usually focused on specific areas and management regimens, using different diagnostic methods, making it difficult to compare results. Information regarding the detection of eggs in feces by coprological techniques shows a highly variable prevalence of *F. hepatica* in sheep throughout Europe. In Spain, a prevalence of 59.3% has been reported [22], in Ireland 50.4% [23], in Poland 10.9% [24], in Italy 7.9%, in Switzerland 4.0% [25] and in Portugal 1.8% [26]. Coprological methods are only sensitive from 8–12 weeks post-infection (wpi), so the presence of the parasite cannot be determined in the acute phase and during pre-patent period of fasciolosis, due to the lack of egg output in feces [27].

As such, antibody detection by ELISA is commonly used to diagnose *F. hepatica* infection due to its high sensitivity on diagnosis and detection of pre-patent infections, when compared with coprological methods [28]. The ELISA method can detect antibodies to *F. hepatica* in serum of experimentally infected sheep from the first wpi [29]. The highest values are obtained between the 4th and 12th wpi, then slowly decreasing until the 32nd wpi [30]. In this study the presence of *F. hepatica* IgG antibodies in sheep was evaluated in two consecutive years. From a total of 92 animals, 18 (19.6%) were seropositive in 2015 and 17 (18.5%) were seropositive in 2016. Nonetheless, we alert that antibody repertoire in naturally chronically infected sheep is not yet well characterized. Natural infections in sheep are typically chronic and last for years, being also associated with repeated exposure [31]. As such, the approach of screening the same animals (prospectively) is likely to reflect a change in the circulation of the parasite. As no seroconversions were observed in this study, one can assume that at least no new infections have occurred in seronegative animals.

Data about *F. hepatica* seroprevalence in grazing sheep in Europe is limited. However, higher results were found in Spain (77.6% in Leon, 56% in Galicia and 42.6% in Pyrenees) [32,33,34], in Sweden (67%) [35] and in Greece (47.3%) [36].

Despite the low seroprevalence values found in the present study, there was one municipality, Gouveia, with a high percentage (62.5%) of seropositive animals in both consecutive years. Interestingly, herds from the Gouveia municipality do not strictly follow the sanitary management defined guidelines regarding deworming for the central region of Portugal. The defined guidelines consider a combination of closantel and mebendazole or ivermectin with clorsulon, known to be effective on adult flukes [20]. Additionally, triclabendazole is also used for effectiveness against young immature liver fluke [37]. No flukicides were ever used in Gouveia municipality, unlike in the rest of the region.

Nonetheless, the resistance of flukes to triclabendazole treatment is becoming a problem [20,37]. The wrong choice of antiparasitic agents, associated with over/underdosage practices at the time of application, may result in the development of resistance. Flukicide resistance was first noted when reporting resistance to triclabendazole in Australia in 1995 [38], later being described in other regions of the world such as Europe [39,40] and South America [41]. Rotational use of triclabendazole, closantel or nitroxynil should be considered where flukicides are used strategically in order to prevent the development of resistance [42]. The *F. hepatica* life cycle is dependent on eco-climatic factors. The extent of rainfall season combined with high average temperature have an influence on the development of *F. hepatica* eggs and larval stages, as well as in the development of its intermediated host [43,44]. Ecological factors such as presence of vegetation [45], poorly drained soils [46], presence of water bodies [47], and management factors as high herd/flock density and absence of flukicide treatment [48], are also important factors that increase the levels of *F. hepatica* infection.

## 5. Conclusions

Albeit a low circulation of *F. hepatica* was found, authors suggest improving practical guidelines for management of fasciolosis in grazing sheep. The correct flukicide treatment is an important strategy to minimize parasite circulation.

## Figures and Tables

**Table 1:** Occurrence of *F. hepatica* IgG seropositive Serra da Estrela sheep sampled in the years 2015 and 2016.

Location	2015 Anti-*F. Hepatica* Positive/Total: No. (%; CI)	2016 Anti-*F. Hepatica* Positive/Total: No. (%; CI)	Seropositivity Differences	*p*
Carregal do Sal	0/12 (0%; 0–26.5)	0/12 (0%; 0–26.5)	0	-
Celorico da Beira	1/28 (3.6%; 0.1–18.4)	1/28 (3.6%; 0.1–18.4)	0	-
Gouveia	10/16 (62.5%; 35.4–84.8)	10/16 (62.5%; 35.4–84.8)	0	-
Seia	7/36 (19.4%; 6.5–32.4)	6/36 (16.7%; 6.4–32.8)	−1	0.76
Total	18/92 (19.6%; 12.03–19.15)	17/92 (18.5%; 11.15–27.93)	−1	0.85

CI, 95% confidence interval.

## Data Availability

Data is contained within the article.
